# Outpatient decolonization after recurrent skin infection with Panton-Valentine leukocidin (PVL)-producing *S*. *aureus—The importance of treatment repetition*

**DOI:** 10.1371/journal.pone.0231772

**Published:** 2020-04-21

**Authors:** Leif G. Hanitsch, Renate Krüger, Pia-Alice Hoppe, Daniel Humme, Anna Pokrywka, Michaela Niebank, Miriam Stegemann, Axel Kola, Rasmus Leistner

**Affiliations:** 1 Institute of Medical Immunology, Charité Universitätsmedizin Berlin, Berlin, Germany; 2 Interdisciplinary workgroup on PVL-positive *S*. *aureus*, Charité Universitätsmedizin Berlin, Berlin, Germany; 3 Department of Pediatric Pulmonology and Immunology, Charité Universitätsmedizin Berlin, Berlin, Germany; 4 Department of Dermatology and Allergy, Charité Universitätsmedizin Berlin, Berlin, Germany; 5 Department of Internal Medicine, Infectious Diseases and Pulmonary Medicine, Charité Universitätsmedizin, Berlin, Germany; 6 Institute of Hygiene and Environmental Medicine, Charité Universitätsmedizin Berlin, Berlin, Germany; Pusan National University, REPUBLIC OF KOREA

## Abstract

**Background:**

Recurrent skin abscesses are often associated with Panton-Valentine leukocidin-producing strains of *S*. *aureus* (PVL-SA). Decolonization measures are required along with treatment of active infections to prevent re-infection and spreading. Even though most PVL-SA patients are treated as outpatients, there are few studies that assess the effectiveness of outpatient topical decolonization in PVL-SA patients.

**Methods:**

We assessed the results of topical decolonization of PVL-SA in a retrospective review of patient files and personal interviews. Successful decolonization was defined as the absence of any skin abscesses for at least 6 months after completion of the final decolonization treatment. Clinical and demographic data was assessed. An intention-to-treat protocol was used.

**Results:**

Our cohort consisted of 115 symptomatic patients, 66% from PVL-positive MSSA and 19% from PVL-positive MRSA. The remaining 16% consisted of symptomatic patients with close contact to PVL-SA positive index patients but without detection of PVL-SA. The majority of patients were female (66%). The median age was 29.87% of the patients lived in multiple person households. Our results showed a 48% reduction in symptomatic PVL-SA cases after the first decolonization treatment. The results also showed that the decrease continued with each repeated decolonization treatment and reached 89% following the 5^th^ treatment. A built multivariable Cox proportional-hazards model showed that the absence of PVL-SA detection (OR 2.0) and living in single person households (OR 2.4) were associated with an independently increased chance of successful decolonization.

**Conclusion:**

In our cohort, topical decolonization was a successful preventive measure for reducing the risk of PVL-SA skin abscesses in the outpatient setting. Special attention should be given to patients living in multiple person households because these settings could confer a risk that decolonization will not be successful.

## Introduction

Recurrent skin abscesses in patients who do not have a predisposing condition are often associated with Panton-Valentine leukocidin-producing strains of *S*. *aureus* (PVL-SA) [1–3]. Although in North America PVL-SA is predominantly associated with community-acquired MRSA related primarily to the clone USA 300 [3, 4], in Europe the vast majority of PVL-positive *S*. *aureus* (PVL-SA) strains are methicillin-susceptible (MSSA) [5]. The verification of PVL-SA colonization or infection requires microbiological screening for *S*. *aureus* and additional PCR testing for the production of Panton-Valentine leukocidin by amplifying the encoding genes *LukS/LukF* [1].

There can be a considerable delay in diagnosis despite its typical clinical presentation with non-immunocompromised patients who suffer from recurrent skin abscesses. This results in multiple episodes of skin infections and often leads to the transmission of pathogens to close contacts [1, 2, 6, 7]. The diagnostic confirmation of a PVL-SA skin infection can be challenging for several reasons. Although microbiological screening has a high sensitivity for monocloncal PVL-positive MRSA, it is not clear how well nasal screening performs in cases of polyclonal MSSA colonization [8–10]. Moreover, often many members of a family or individuals in the same household can be affected. But the ping-pong-like transmission and retransmission events of *S*. *aureus* can make PVL-SA colonization time-dependent [10, 11]. This makes it difficult to eradicate PVL-SA colonization in all potential carriers.

After the primary treatment of PVL-SA-related infections, secondary prevention requires topical decolonization [6, 12–14]. Despite the fact that many patients show relevant skin infections, most cases can be treated in an outpatient setting. This includes decolonization procedures. However, there is a dearth of studies regarding outpatient decolonization and its specific hurdles in the outpatient setting for patients with recurrent PVL-SA skin infections. Hence, in this article we will focus on factors for the success of PVL-SA topical decolonization that are relevant to the outpatient setting.

## Methods

Our cohort consisted of patients with recurrent skin infections combined with the detection of PVL-positive S. aureus (PVL-SA) as well as their close contacts who sometimes also displayed symptoms. All cohort members were seen in our outpatient clinic between December 2010 and August 2017. Recurrence of an abscess was defined as more than one abscess that required surgical drainage within a two-month period. Patients with a (predisposing) chronic skin condition, e.g. Acne inversa or severe atopic dermatitis, were excluded from the present study. In clusters with more than one symptomatic and / or PVL-SA-positive patient, the topical decolonization protocol was followed for all household members. In case of active PVL-SA skin infections, patients received a rifampicin-based dual antibiotic regime combined with TMP/SMX or as determined by antibiogram results. In addition, patients were advised to start a decolonization treatment at home after the resolution of the infection. Decolonization involved a 5-day standard decolonization protocol that included daily antiseptic washing of the whole body and hair with a commercially available octenidin-based product, antiseptic treatment of the oral cavity (octenidin-based), intranasal application of mupirocin, and extended hygiene measures including the washing of clothes at more than 60°C and disposing of personal care products [2].

Success of the decolonization procedure was defined as a period of at least six months without a skin abscess or other SSTI following completion of the decolonization treatment. Decolonization was repeated in cases when skin abscesses recurred. The following variables were systematically evaluated in interviews to assess the factors that reduced the success of decolonization: age, gender, number of decolonization treatments, household size, number of close contacts to the index case, abscess localization, and phenotype of PVL-SA (MRSA or MSSA). A cluster was defined as individuals in the index patient’s immediate environment (e.g. family or friends with frequent close physical contact) who were also colonized or infected with PVL-SA. Household size was defined as the number of individuals living together in a household with the index patient.

All patients were screened for PVL-SA in nares, throat and wounds, if applicable. Swabs were cultivated on Columbia Agar with 5% sheep blood. Species identification and antimicrobial susceptibility testing were performed using a Vitek 2 system and applying EUCAST breakpoints. The detection of PVL *LukS/LukF* was performed using PCR [15].

In order to assess parameters influencing the length of time needed for successful decolonization, we performed a univariate Kaplan-Meier analysis and a multivariable Cox regression of all symptomatic patients, applying an intention-to-treat protocol. All analyses were performed using SPSS (IBM SPSS statistics, Somer, NY, USA).

Ethics approval and consent to participate: The study was conducted in accordance with the Declaration of Helsinki, national, and institutional standards. The study was approved by the local Ethics Committee (Charité, Berlin, Germany, EA2/190/17). The study is based on secondary data that was generated for routine clinical care. Within this scope, written consent for treatment purposes and secondary analysis was provided by each patient. Consent for minors under age 18 was obtained from parents or guardians. The Ethics Committee waived the requirement for additional consent for this study.

## Results

**Fig 1:**
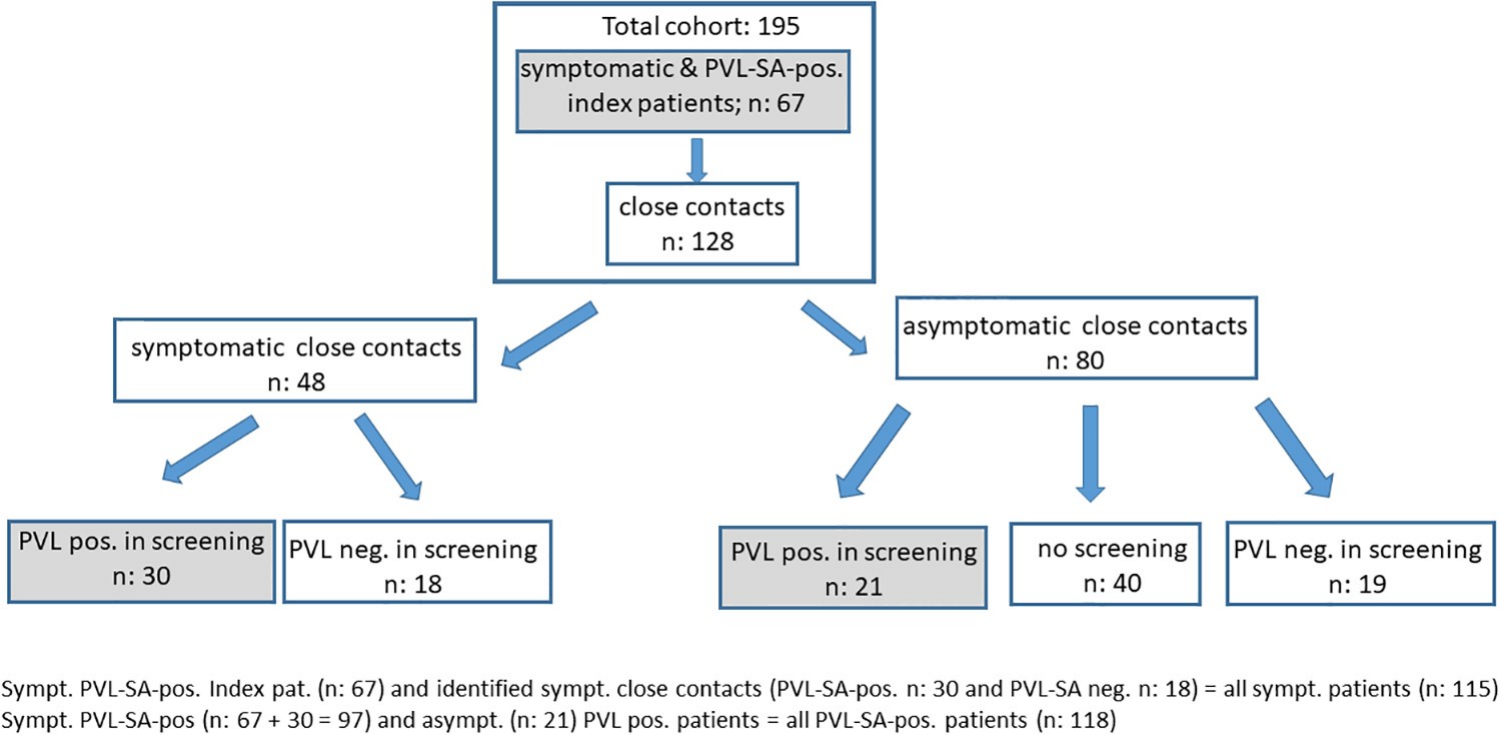
Flow chart of all enrolled patients.

Sixty seven index patients with PVL-SA-positive recurrent skin abscesses presented to our outpatient clinics between December 2010 and August 2017. Altogether, they reported 128 close contacts (accounting for n: 195). 48 of 128 contacts reported recurrent skin abscesses. Of the remaining 80 asymptomatic contacts, 40 contacts participated in PVL-SA screening, revealing an additional 21 PVL-SA positive cases. The remaining 40 asymptomatic contact patients were not available for examination of PVL-SA (Fig 1).

Taken together, of 155 patients and contacts, PVL-SA was verified in 118 cases (76%). One patient was colonized with both phenotypes, hence 27 MRSA (23%), 92 MSSA (78%) were detected.

By the time of diagnosis (skin abscess due to PVL-SA), the patients’ medical histories showed a median of 4 episodes of skin abscesses (IQR 2–10) and a median delay of 5 months between first episode and final diagnosis (IQR 1–14). We found it helpful to calculate the average number of abscesses per month as an estimator of the severity of the infection and the psychological strain on patients. The result was a median of 1.0 (IQR 0.3–2.8). Common abscess sites included extremities (50%), trunk (47%), and head or face (42%), without any specific distribution pattern. Few patients (4%) showed invasive infections, such as necrotizing fasciitis, necrotizing pneumonia or bloodstream infection.

Based on the patients’ past medical histories, many reported that they had undergone multiple antibiotic treatments and multiple episodes of surgical intervention before being diagnosed with skin abscesses from PVL-SA. 38 (33%) patients reported hospital stays for the treatment of their recurrent abscesses with a length of stay between 1 and 42 days (median 3 days). Table 1 provides an overview of all assessed parameters.

**Table 1:** Overview on the analyzed PVL-SA patients.

Parameter		Total cohort (n = 155)	Clinically symptomatic (n = 115)	Clinically asymptomatic (n = 40)
		Median (range) / % (number)	Median (range) / % (number)	Median (range) / % (number)
Age (years)		29 (0–75)	28 (0–65)	31 (0–75)
Male sex		44% (68)	43% (49)	48% (19)
Months before diagnosis		not applicable	5 (0–158)	not applicable
Number of abscesses before diagnosis		not applicable	4 (1–100)	-
Number of antimicrobial treatments before diagnosis		1 (0–15)	1 (1–15)	0 (0–3)
Number of surgical treatments before diagnosis		not applicable	1 (1–13)	-
Detected pathogen PVL-positive *S*. *aureus*	MRSA	17% (27)	19% (22)	13% (5)
	MSSA	59% (92)	66% (76)	40% (16)
	Not detected	24% (37)	16% (18)	48% (19)
Successful decolonization	Yes	88% (137)	89% (102)	not applicable
Lost to follow up		8% (13)	11% (13)	-
Number of decolonization treatments		1 (1–5)	1 (1–5)	1 (1–3)
Household size	Single person	10% (15)	13% (15)	-
	Multiple persons	90% (140)	87% (100)	100% (40)
Abscess Localization	Extremities	37% (58)	50% (58)	-
	Trunk	35% (54)	47% (54)	-
	Head/Face	31% (48)	42% (48)	-
	Gluteal	22% (34)	30% (34)	-
	Axilla	13% (20)	17% (20)	-
	Genital	11% (17)	15% (17)	-
	Inguinal	5% (8)	7% (8)	-
	Invasive	3% (4)	4% (4)	-

MRSA, methicillin-resistant *S. aureus*. MSSA, methicillin-susceptible *S. aureus*. PVL, Panton-Valentine leukodicin. Successful decolonization was defined as remaining clinically asymptomatic for at least 6 months after final decolonization treatment.

**Fig 2:**
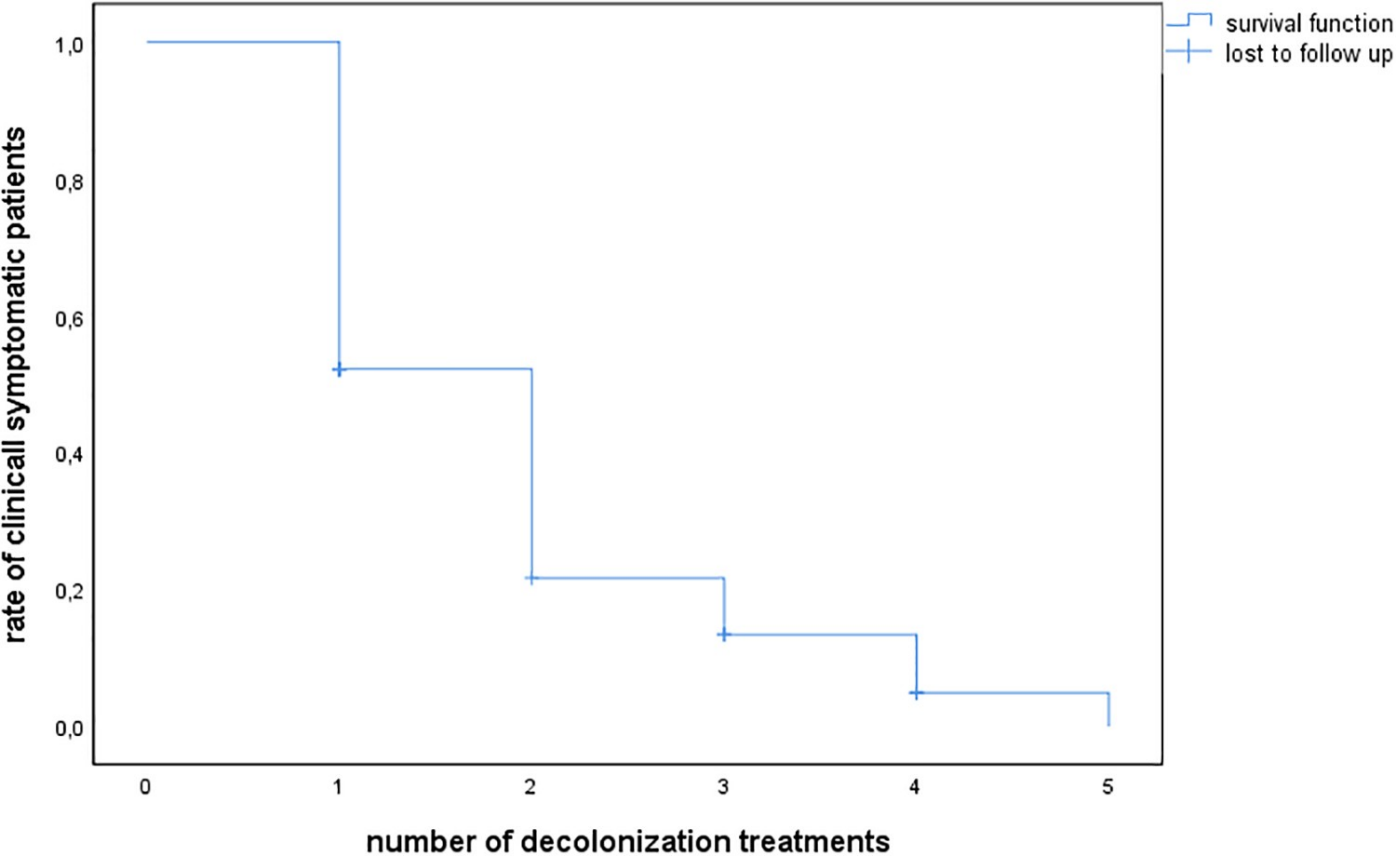
Kaplan-Meier curve of PVL-SA decolonization in 115 symptomatic patients. PVL-SA, Panton-Valentine leukocidin-positive *Staphylococcus aureus*.

Following an intention-to-treat protocol, we analyzed all 115 symptomatic patients for successful topical decolonization. 77 (67%) received systemic antimicrobial treatment beforehand without relevant reduction in infection incidence. The median follow-up time was 15 months after the final decolonization treatment. After the first decolonization treatment, the number of symptomatic patients was reduced by 48% (56 patients) (Fig 2). 13 patients were lost to follow up (11%). Altogether, this amounted to a success rate of 89% after 5 decolonization treatments.

**Fig 3:**
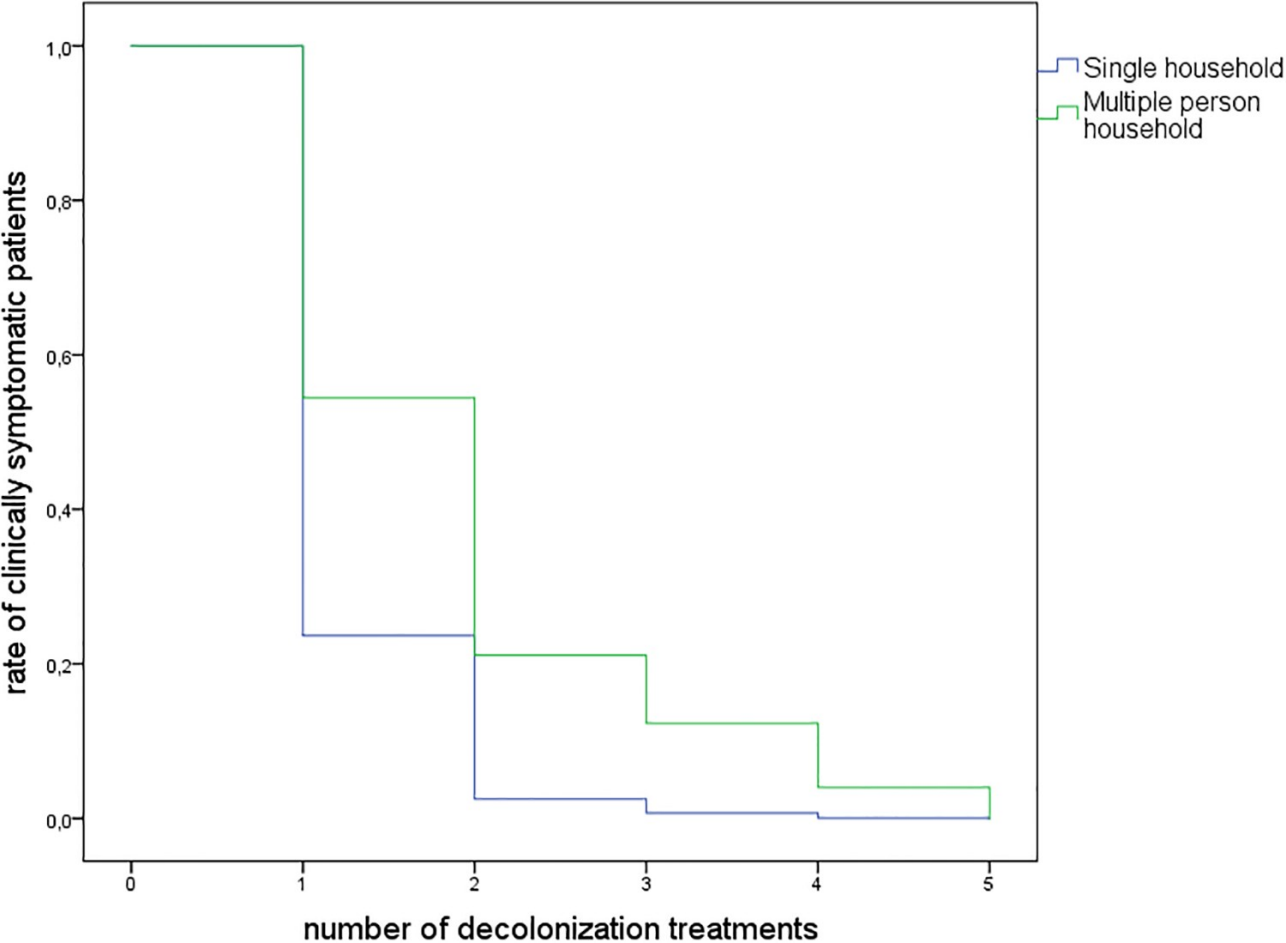
Multivariable Cox regression curve of PVL-SA decolonization in 115 symptomatic patients. PVL-SA, Panton-Valentine leukocidin-positive *Staphylococcus aureus*.

The following parameters were considered in the Cox regression analysis: age, gender, number of decolonization treatments, single vs. multiple person household and phenotype of PVL-SA (MSSA/MRSA). Factors independently associated with a successful decolonization were living in a single household (in contrast to a multiple person household) and undetected PVL-SA in symptomatic patients (Table 2 and Fig 3).

**Table 2:** Multivariable Cox regression analysis of parameters associated with successful decolonization.

Parameter	P-value	OR	95% Confidence Interval	
			Lower	Upper
PVL-S. aureus phenotype				
MSSA	Reference			
MRSA	0.615	0.873	0.516	1.479
Undetected	0.012	2.030	1.170	3.523
Single household	0.006	2.372	1.285	4.381

PVL, pantone-valentine leukocidine. MSSA, Methicillin-susceptible *S. aureus*. MRSA, methicillin-resistant *S. aureus*. OR, odds ratio.

## Discussion

Skin and soft tissue infection caused by PVL-positive *S*. *aureus* are frequently associated with recurrent episodes of infection, delayed diagnoses, and eventually an increased risk of transmission often resulting in clusters of affected patients in close personal proximity [1–3, 6, 16]. Although these infections are often community-acquired, there is a lack of literature that analyzes the effectiveness of outpatient decolonization [17, 18].

Pathogen transfer within households has been described repeatedly as the dominant transmission route for community-acquired *S*. *aureus* in the past [7, 11, 19, 20]. Our data underscores this observation for PVL-SA, as we found that 69/88 (78%) contacts screened were also clinically symptomatic and / or PVL-positive (Fig 1). The clinical relevance of household contact is further emphasized by our observation that living in a multiple person household was a relevant risk factor for requiring repeated decolonization as compared to living alone.

Knox et al. stated that the degree of physical contact among household members and the amount of time spent at home determine the risk of transmission [11]. This could result in epidemic *S*. *aureus* clones ‘ping-ponging’ between family members [19–22]. These observations support the need for the simultaneous decolonization of close physical contacts, at least in cases where patients remain PVL-SA colonized despite decolonization treatment.

Our results show that symptomatic patients in which PVL-SA could not be detected have a higher chance of successful decolonization. Hence, this observation could be an indication that a low-level colonization with PVL-SA might facilitate the eradication process. Earlier studies of MRSA decolonization have shown that the success of eradication can be dependent on the localization of the colonization [23–25]. In our cohort, testing for PVL-SA was not performed separately, using different swabs for nose and throat.

In the present study many patients reported a past medical history typical of PVL-SA patients–recurrent skin abscess that often required incision and drainage and reporting other affected patients in their immediate environment. PVL-SA-positive patients are often symptomatic for several months, before they are finally diagnosed and treated for PVL-SA [16, 26–29]. Our results thus underscore earlier work that shows that a diagnosis can be challenging in areas with low PVL-SA prevalence [6, 30].

The patients in our cohort were on average 29 years old, confirming earlier studies on patients with community-onset PVL-SA-associated skin abscess [30, 31]. Whether there are socio-cultural or medico-biological factors driving this observation is unclear.

Our study has several limitations. This is a retrospective study on PVL-SA patients from a single institution. It represents the patient population from our area and mirrors the results of our locally established treatment procedures. All patients with an active infection were treated with an oral antimicrobial agent—at least once—parallel to topical treatment. This makes it impossible to determine the effect of the antimicrobial therapy on the decolonization results. However, considering the fact that 2/3 of our patients had received antibiotics without any topical decolonization and continued to suffer from recurrent skin abscesses argues against a major protective role of isolated antibiotic use in these patients. Patients in our cohort had a mean total of four skin abscesses in the five months prior to topical decolonization; after successful decolonization, all patients were asymptomatic for at least 6 months with a mean follow-up of 15.6 months.

## Conclusion

Our data shows that patients with skin abscesses associated with PVL-SA can be successfully treated with decolonization as outpatients, but they often require multiple attempts at decolonization. The delayed success of treatment of patients living in multiple person households suggests that decolonization treatment should be conducted simultaneously on all close contacts.

## Supporting information

S1 Database (XLSX)

## Abbreviations

IQR: interquartile range
MRSA: Methicillin resistant *Staphylococcus aureus*
MSSA: Methicillin susceptible *Staphylococcus aureus*
PCR: polymerase chain reaction
PVL: Panton-Valentine leucocidin
SA: *Staphylococcus aureus*
